# Implications for End-of-Life Care: Comparative Analysis of Advance Directives Laws in Taiwan and the United States

**DOI:** 10.1177/10499091251328007

**Published:** 2025-03-24

**Authors:** Yufang Tu, Yuchi Young, Melissa O’Connor

**Affiliations:** 1 Department of Human Development and Family Science, 3323 North Dakota State University, Fargo, ND, USA; 2 Department of Health Policy, Management, & Behavior, 1084 University at Albany, Rensselaer, NY, USA

**Keywords:** advance directives, advance care planning, advance directives laws, palliative medicine, end-of-life care, cross-cultural comparation, Patient Self-Determination Act (PSDA), Patient Right to Autonomy Act (PRAA)

## Abstract

This study explores end-of-life care decisions across cultures by comparing advance directives (ADs) laws in the United States (U.S.) and Taiwan. Specifically, it examines the U.S.’s 1991 Patient Self-Determination Act (PSDA) and Taiwan’s 2019 Patient Right to Autonomy Act (PRAA). By analyzing key legal differences and similarities, the study provides insights into improving end-of-life care policies and understanding how legal frameworks shape patient autonomy globally. This review utilized the keywords “United States or Taiwan,” “Patient Self-Determination Act,” “Patient Right to Autonomy Act,” “advance directives,” and “advance care planning,” with searches restricted to English or Chinese publications since 1991. The analysis shows that both the U.S. and Taiwan view ADs as crucial for healthcare autonomy, enabling individuals to make decisions in advance and allowing healthcare agents to act on their behalf if they become incapacitated. However, ADs laws differ notably in their requirements, scope, completion processes, healthcare agent eligibility, portability, and promotional efforts. In the U.S., while various types of ADs are available (e.g., MOLST, POLST, Five Wishes), stricter regulations are needed to govern interactions between patients and healthcare agents to ensure that healthcare decisions align more closely with patients’ preferences. Improving AD portability, particularly in emergencies, through cross-state recognition or digital sharing, is essential. For Taiwan, recommendations include enhancing palliative care practices and expanding ADs to include emotional and spiritual preferences. Integrating psychiatric ADs into Taiwan’s PRAA could provide significant benefits. Additionally, reducing the costs associated with advance care planning and increasing AD awareness through active healthcare involvement would further strengthen patient autonomy.

## Introduction

Medical advances have extended life expectancy and improved quality of life, even for those with chronic illnesses.^1^ These advances have blurred life and death boundaries, possibly extending suffering.^1^ The final stage often lacks dignity and autonomy, making it a painful ordeal. Consequently, significant research has been conducted into advance care planning (ACP) and advance directives (ADs) to empower individuals to manage their own end-of-life care. ADs are vital to aligning end-of-life healthcare decisions with personal values.^2,3^

An AD outlines medical treatment preferences if one becomes unable to communicate.^3-6^ These documents legally uphold individual healthcare autonomy.^3,6^ ADs outline preferences for end-of-life care if decision-making capacity is lost.^6^ Many developed countries, including the United States (U.S.) and Taiwan, have laws protecting patient autonomy in healthcare decisions, ensuring person-centered care.^7^

The Patient Self-Determination Act (PSDA) was enacted federally in the U.S. in 1991.^1,8^ It legally recognizes the importance of ADs and encourages their use to safeguard individuals’ freedom in determining medical treatments.^1,2,8,9^ The Act protects patient autonomy concerning life-sustaining treatments (LST) under conditions such as terminal illness, persistent vegetative state, or severe dementia.^6,9,10^ Likewise, Taiwan’s Patient Right to Autonomy Act (PRAA), effective in 2019, marks the first legislation among Asian countries to honor and promote medical autonomy, ensuring the right to a dignified death and fostering a harmonious physician-patient relationship.^11-13^ The PRAA allows patients to refuse LST or artificial nutrition/hydration in conditions like terminal illness, irreversible coma, vegetative states, severe dementia, or other incurable diseases causing persistent pain.^14^

Although both countries’ legislation shares similar purposes, their differences merit analysis to encourage mutual learning. Cross-cultural comparisons like the current study are essential for enhancing understanding of the role of cultural norms in improving healthcare practices, fostering international collaboration, serving culturally diverse individuals, and promoting internal innovations to inform policy development.^15-17^ The current study aims to explore these legal distinctions and their implications for end-of-life care through a comparative analysis by addressing 2 research questions: (1) what are the legal differences in ADs laws between the U.S. and Taiwan? and (2) what can each learn from the other to enhance end-of-life care practices? This study discusses the legal frameworks of the U.S.’s PSDA and Taiwan’s PRAA, exploring their key components, legal requirements, participants required in the process, and promotional efforts in completing ADs. The analysis outlines ADs laws’ differences and suggests policies to improve end-of-life care. This study concludes with limitations, future research directions, and final thoughts, emphasizing how the findings can inform research, practices, and policy.

## Methods

To identify relevant literature regarding the PSDA, ADs, and ACP, we searched the ProQuest database (Table 1); Congress Report; federal and state government websites on health services (e.g., Medicaid, Medicare, hospice and palliative care, ADs, and ACP); and legal, policy, and healthcare websites related to ADs and ACP (e.g., American Bar Association, Aging with Dignity). The selection in ProQuest was based on English peer-reviewed scholarly journals published in the U.S. since 1991, with the following keywords: Patient Self-Determination Act, advance directives, and advance care planning.

**Table 1.** Identification of Studies on the United States’ Patient Self-Determination Act (PSDA) via the ProQuest Database.

Identification: ProQuest database (n=48)
↓
Screening & Eligibility: Abstract Assessed for Eligibility
• Keywords: Patient Self-Determination Act, advance directives, and advance care planning.
• Limitation to search: peer reviewed
• Source type: scholarly journals
• Publication date: December 1 1991 to August 31 2024
• Subject (include): advance directives
• Region: The United States
• Language: English
↓
Excluded (*n* = 40) (Articles targeting a specific ethnic group or focusing on a specific case study were excluded, as they were not directly relevant to our current research.)
• Nursing home residents: 7
• Patients with specific illnesses or belonging to certain health-related groups (e.g., seriously ill hospitalized patients, seriously ill patients, hospital patients, healthcare providers, incapacitated patients, hastened death cases, xenotransplantation recipients, users of implantable devices, and those undergoing life-sustaining treatment, or experiencing early deaths): 10
• Studies on certain groups regarding AD requirements/elements (e.g., healthcare proxy, witness, code status of ADs, outcomes of surrogate decision-making): 4
• Specific groups based on age or race/ethnicity (e.g., adolescents, homebound older adult clients, Bosnian immigrants, racially/ethnically diverse groups): 4
• Ethical and legal issues or use in artificial nutrition/hydration: 3
• CComparative studies (e.g., PSDA vs. Uniform Health Care Decisions Act; ADs across states): 2
• Orders (e.g., do-not-resuscitate orders, do-not-hospitalize orders): 2
• Others (e.g., preface of reports, case studies, AD promotion program evaluations, improving/reforming end-of-life care, multicultural/ethnic considerations, conscience rules): 7
• Duplicate: 1
↓
Included (n=8) (Articles included in this review for content analysis and comparison between the PSDA and PRAA)

*Note*: PRISMA flowchart (illustrating the search process). Reference: PRISMA. (2024). *Welcome to the new Preferred Reporting Items for Systematic Reviews and Meta-Analyses (PRISMA) website*. https://www.prisma-statement.org/

To identify relevant literature regarding the PRAA, ADs, and ACP, we searched the ProQuest database (Table 2); the Hospice Foundation of Taiwan; the Taiwan Laws & Regulations Database, the Taiwan Ministry of Health and Welfare; and other news articles for the latest policy and information releases as needed. The selection in ProQuest was based on English and Chinese peer-reviewed scholarly journals published in the U.S. and Taiwan since 2019, with the following keywords: the Patient Right to Autonomy Act, advance directives, and advance care planning.

**Table 2.** Identification of Studies on Taiwan’s Patient Right (PRAA) to Autonomy Act via the ProQuest Database.

Identification: ProQuest database (n=34)
↓
Screening and Eligibility: Abstract Assessed for Eligibility
• Keywords: Patient Right to Autonomy Act, advance directives, and advance care planning.
• Limitation to search: peer reviewed
• Source type: scholarly journals
• Publication date: January 1 2019 to August 31 2024
• Subject (include): advance directives
• Region: Taiwan
• Language: English and Chinese
↓
Excluded (n = 31) (Articles targeting a specific ethnic group or focusing on a specific case study were excluded, as they were not directly relevant to our current research.)
• Nursing home residents: 2
• ACP/ADs/palliative care services program or questionnaire testing: 3
• Case, pilot, or program studies: 4
• Specific groups (e.g., caregivers, inpatient family members): 2
• Health workers/Taiwanese nurses: 2
• Attitudes and preferences for life-sustaining treatment: 2
• Personality traits related to completing ADs: 1
• Patients with specific illnesses (e.g., cognitive impairment, mild dementia, cancer, terminal cancer, chronic life-limiting illness, hemodialysis, terminally ill, psychiatric patients, renal disease): 14
• Duplicate: 1
↓
Included (*n* = 3) (Articles included in this review for content analysis and comparison between the PSDA and PRAA)

*Note*: PRISMA flowchart (illustrating the search process). Reference: PRISMA. (2024). *Welcome to the new Preferred Reporting Items for Systematic Reviews and Meta-Analyses (PRISMA) website.* https://www.prisma-statement.org/

## Results

### Legal Frameworks of the PSDA and PRAA

#### The U.S.'s PSDA

The Patient Self-Determination Act (PSDA) was enacted in December 1991.^18,19^ The PSDA promotes using ADs to respect and implement patients’ end-of-life healthcare preferences.^1^ The PSDA has 2 main components: a living will that outlines an individual’s medical treatment preferences and the appointment of a healthcare agent (durable power of attorney for healthcare) to make decisions if the individual is unable to do so.^1,2,20^ The PSDA aims to protect and implement patient autonomy and person-centered healthcare by urging competent adults to complete ADs.^2,7^ The Act requires healthcare facilities participating in Medicare and Medicaid to ask patients about ADs, provide relevant information, and document ADs-related details in their medical records.^1^

An AD can be completed by an individual, often with assistance from healthcare professionals or a lawyer. The PSDA does not require the involvement or consent of family members or medical professionals for ADs consultation or certification. The rules and requirements for ADs, such as eligibility, age, witnesses, and notarization, vary by state. Generally, individuals aged 18 and older can complete an AD independently, though some states may require a witness or notary to validate the document’s legal standing.^1,21^

The U.S. PSDA mandates that healthcare professionals honor patients’ decisions as outlined in their ADs.^20^ Although there have been cases where courts ruled in favor of hospitals despite failures to honor a patient’s AD, leading to inappropriate LST, such as *Cronin v. Jamaica Hospital Medical Center (NY App Div, 2009)*, more recent cases indicate a shift toward holding hospitals accountable.^22^ For instance, in *Koerner v. Bhatt (NJ Super Ct Law Div, Morris Cty, 2017)*, a New Jersey court found the hospital liable for administering unwanted life support in violation of the patient’s documented Do-Not-Intubate and Do-Not-Resuscitate (DNR) orders.^23^ This case underscores the legal obligation of healthcare institutions to respect a patient’s end-of-life preferences. Other cases, such as *Doctors Hospital of Augusta v. Alicea Administratrix (2016)*^24^ and *Weisman v. Maryland General Hospital, Inc.*,^25^ further demonstrate hospitals’ liability for violating patients’ ADs.

#### Taiwan's PRAA

The Patient Right to Autonomy Act officially came into effect in January 2019 in Taiwan.^26^ The PRAA outlines the legal framework for advance medical directives, consisting of 3 parts. First, ACP consultation involves individuals, their family, or others, along with healthcare professionals. Second, an advance medical decision is a formal written document expressing medical wishes, allowing individuals to refuse or receive treatments under specific conditions. Third, individuals can appoint a healthcare agent (though not mandatory), designating a trusted adult to make healthcare decisions on their behalf if they become incapacitated.^27^

The PRAA focuses on patients with incurable diseases and their decisions regarding life-prolonging treatments.^14^ It applies to 5 categories: terminal illness, irreversible coma (after 3-6 months with no signs of consciousness recovery),^28^ vegetative state (3-6 months with no improvement),^29^ severe dementia (inability to perform self-care or work, diagnosed with high Clinical Dementia Rating or Functional Assessment Staging Test),^30^ and other incurable diseases causing severe pain (as announced by the government).^14^ The Ministry of Health and Welfare has listed 12 rare diseases, such as cystic fibrosis and amyotrophic lateral sclerosis, which differ from the above categories in disease progression.^31,32^

The Ministry of Health and Welfare regularly seeks input from patient groups, healthcare institutions, medical associations, and experts on diseases or conditions applicable to the other-disease category under Article 14 of PRAA.^14^ After announcing the list, the Ministry collaborates with rare-disease groups to support ACP processes and ADs completion, ensuring patients’ medical wishes are honored and their right to a good death.^32^

Patients can exercise autonomy over specific medical treatments, including LST (e.g., cardiopulmonary resuscitation, mechanical support, blood transfusions, treatments for specific diseases, and antibiotics for severe infections) and tube-feeding or other invasive nutritional/hydration methods.^33,34^

The PRAA, specifically Article 14 Section 5, holds medical providers accountable for damages if they violate a patient’s AD.^14^ Providers who knowingly disregard these instructions can be held liable for any resulting harm. Taiwan’s legal system is based on a continental law tradition, unlike the U.S. common law system, where court decisions shape future rulings. In Taiwan, the application of written regulations or codes determines legal outcomes. While no court decisions in Taiwan have yet directly addressed breaches of PRAA Article 14 Section 5, the written code serves as the foundation for determining acceptable healthcare practices, emphasizing the importance of respecting patients’ documented wishes in ADs as fundamental to patient rights and decision-making.^35^

### Similarities in Legal Framework Between the PSDA and the PRAA (Section A & Section B)

Both the US and Taiwan share common principles in advocating ADs, recognizing them as legal documents to uphold individual autonomy in healthcare decision-making. They allow individuals to accept or decline treatments, including life-sustaining measures, and encourage expressing medical wishes through ADs. Both the PSDA and PRAA permit the appointment of a healthcare agent or advocate to make decisions on behalf of an incapacitated individual (Table 3).

**Table 3.** Comparative Analysis of Advance Directives Legal Frameworks Between the United States and Taiwan.

	The United States The Patient Self-Determination Act (PSDA)	Taiwan The Patient Right to Autonomy Act (PRAA)
A. The Purpose of ADs Laws in Protecting Patient Autonomy in Healthcare Decision-Making	Both the United States (U.S.) and Taiwan regard advance directives (ADs) as legal documents designed to respect and protect individual autonomy in medical decision-making, such as accepting or refusing life-sustaining treatment (LST), resuscitation, and other medical treatments. Laws regarding ADs also encourage individuals to express and formulate their medical wishes through ADs and require health professionals to honor and respect individuals' medical decisions.	
B. Appointments of Healthcare Agents Under ADs Laws	ADs laws in both the U.S. and Taiwan provide the option of appointing a healthcare agent or advocate (e.g., a family member or trusted person) to make medical decisions on the individual's behalf if one becomes incapacitated.	
C. The Laws Concerning ADs and Patient Autonomy	The U.S.’s PSDA officially came into effect in December 1991. The PSDA is a federal law that establishes patients' rights to make healthcare and medical-related decisions and to promote the use of ADs.	The Taiwan’s PRAA officially came into effect in January 2019. This Act stipulates the legal effect of advance medical decisions. It marks the first legislation among Asian countries to honor and promote medical autonomy, ensuring the right to a dignified death and fostering a harmonious physician-patient relationship.
D. The Scope and Components of ADs Laws	ADs is a general term for various documents and includes the following three parts. The PSDA emphasizes the first two parts: 1. Living Will: The patient expresses his/her medical wishes in writing. It is a directive that expresses a person's wish to receive or deny healthcare treatments (e.g., LST, resuscitation, artificial nutrition, or other medical interventions) when a person cannot make decisions. It would take effect only if certain medical criteria are met in most states, such as being unable to make medical decisions or communicate wishes. 2. Appointment of a healthcare agent (durable power of attorney for healthcare): The patient designates an agent to make medical decisions on his or her behalf when the patient cannot communicate and make medical decisions. 3. Specific wishes (e.g., Five Wishes): It allows individuals to state their healthcare preferences and values (e.g., emotional or spiritual aspects) that are important to them.	PRAA consists of three parts: 1. Advance care planning (ACP) consultation: A counseling process involving the individual declarant, his or her family members or other relevant parties, and healthcare professionals. 2. Advance decision: A formal written document of an individual's medical wishes that allows the individual to refuse or receive certain medical treatments (e.g., LST, artificial nutrition/hydration) under certain circumstances. 3. Appointment of a healthcare agent: Though not mandatory, the patient has the right to designate an agent to make medical decisions on his or her behalf when the patient cannot communicate and make medical decisions.
E. The Inclusiveness of ADs: Addressing Medical, Emotional, Spiritual, and Mental Aspects	Five Wishes is a legally binding ADs form that covers the personal, emotional, and spiritual aspects of end-of-life care, allowing individuals to document their wishes and preferences holistically. Additionally, the PSDA includes Psychiatric Advance Directives (PADs), which provide individuals with mental illness with a legal mechanism to make decisions about future psychiatric treatment and hospitalization before an emergency occurs.	According to the PRAA, although once an AD is initiated for a patient to terminate or withdraw LST-related treatments, palliative and supportive care will be provided to relieve the patient's physical and mental suffering, the ADs format is primarily focused on documenting the individual's medical preferences, with limited emphasis on emotional and spiritual aspects.
F. Participants Required for Completing an AD	An individual declarant, an attorney, or another person can complete an AD. The PSDA does not require the consent or the company of one's family member in the process, nor does it require ACP consultation from a healthcare professional, though this may vary by state. The rules and requirements for filling out an AD (e.g., who can fill it out, age requirements, presence of witnesses, or notarization of AD documents) vary by state. In general, individuals 18 years of age and older can complete an AD on their own and are not required to be accompanied by others. However, some states or jurisdictions may require that the document be signed in the presence of a witness or notary confirming their legal effectiveness.	The PRAA requires the presence of a legally qualified individual declarant (20 years and older or married) and at least one family member to conduct an ACP consultation with a designated healthcare professional in advance before signing/filling out an AD. Individual declarants are responsible for paying the ACP consultation fee (USD $100/hour) at their own expense. By the end of 2024, four categories of patients (described below) are expected to receive ACP consultation fee waivers, potentially benefiting approximately 0.2 million people in Taiwan: 1. People who are terminally ill 2. People diagnosed with mild dementia, 3. People diagnosed with rare diseases recognized by government agencies, and 4. People with limited mobility or disability but with an independent will.
G. Eligibility Criteria for Healthcare Agents	The PSDA does not specify the relationship between the patient and the designated healthcare agent. It refers to the designation of a competent adult whom the patient trusts and understands their values and preferences to make decisions on their behalf. Some states (e.g., North Dakota) may impose restrictions, such as prohibiting healthcare or long-term care providers or their employees from being designated as a patient's healthcare agent, but do not include restrictions involving the types of conflicts of interest that should be avoided between patients and their healthcare agent.	The healthcare agent should be a competent adult with written consent. Exclusions: Other than being an heir, the healthcare agent cannot be the patient’s legatee, the designated recipient of the patient’s remains or organs, or someone benefitting from the patient’s death.
H. The Portability of ADs	A cloud-based sharing system for medical records across states or medical institutions is still lacking, posing a challenge to the cross-state portability of ADs. However, some organizations (e.g., ADVault, Inc., Thorough Care) provide online services that allow individuals to share and upload their AD forms (e.g., Five Wishes, POLST, or MOLST forms) or ACP documents, including audio or video messages, to their free online accounts.	Under Taiwan's PRAA, completed and signed AD documents must be registered and marked on the individual's National Health Insurance Card. With the individual's informed consent, their medical records, including AD documentation, are accessible in the cloud and visible to all healthcare institutions nationwide in Taiwan.
I. Strategies for Promoting ADs	Upon admission to a healthcare facility, patients receive written notice of their rights to make medical decisions, refuse medical care, and create ADs for healthcare. Healthcare facilities under Medicare or Medicaid must ask patients about their rights to make medical decisions (informed consent and refusal of treatment) and provide written information about these rights, including the right to create ADs. The facility must document the patient's ADs in their medical record.	ACP and AD information is released to the public on a voluntary basis by medical institutions, and news media. However, additional channels are needed to effectively communicate these messages and increase awareness of ADs.
J. Palliative Care’s Role in ADs	Palliative care (PC) teams facilitate the completion of ADs, enhancing the quality of life for patients and families facing serious illnesses. They lead ACP discussions, promote patient-centered decision-making, navigate legal and ethical complexities, educate healthcare professionals, and offer emotional support during end-of-life planning.	PC teams are crucial in developing and completing ADs, enhancing the quality of life for patients in their final stages. For terminally ill inpatients, physicians arrange consultations with PC teams to assist in the development of ADs, particularly for end-of-life decisions. Outpatient home care services involve a multidisciplinary team that visits patients regularly. Healthcare institutions support AD completion through social workers, nursing staff, and trained volunteers, ensuring clear documentation of patients' end-of-life wishes.

### Differences in Legal Framework Between the PSDA and the PRAA (Section C – Section J)

However, the legal frameworks of ADs in the U.S. and Taiwan differ in key aspects, including the legal components of ADs, the scope of ADs law, the steps for ADs completion, healthcare agent eligibility, and the portability and promotion of ADs (Table 3).

#### Legal Components for Completing an AD: Legalization of ACP Consultation (Section C & Section D)

In contrast to Taiwan’s PRAA, which mandates an ACP consultation before completing ADs, the U.S. PSDA does not require such consultations. In the U.S., healthcare professionals simply ask patients if they have AD documents and provide written information, without further communication about the details of the ADs.^21^ Since January 2016, Medicare providers are reimbursed for offering voluntary ACP consultations during annual wellness visits, at no cost to patients. This incentivizes healthcare facilities to share AD-related knowledge with patients and involving members.^36^ If ACP consultations occur outside one’s yearly wellness visit or are deemed part of one’s medical services, costs may be induced on the patient’s side (e.g., deductible or coinsurance may apply).^36,37^ While patient participation in ACP consultations is recommended, the process may proceed without the patient, involving only qualified healthcare professionals and family members or healthcare agents.^36^ Studies show that telehealth ACP is feasible and eligible for coverage under telehealth services. It helps facilitate ACP or AD completion and reduces related medical costs.^36,38-40^

In Taiwan, under the PRAA and related policies, individuals must contact hospitals offering ACP services to consult with a team comprising a trained physician, a nurse, and a psychotherapist or social worker.^11,41,42^ Healthcare institutions in remote areas or with specific expertise can apply to the local health bureau to provide ACP consultation services. Rural health institutions have relaxed requirements for ACP teams, needing at least 1 physician and 1 nursing staff, psychotherapist, or social worker.^41^ The patient or declaring individual must attend the ACP consultation accompanied by at least 1 relative or family member, such as a first-degree relative (spouse, parents, or children) or a second-degree relative (siblings, grandparents, or grandchildren).^41,43,44^

Individuals are responsible for paying their ACP consultation fee (USD $100/hour) on their own, with costs varying by healthcare institutions.^41,42^ However, low- and middle-income households and individuals with disease conditions announced by the governemnt are exempt from the ACP consultation fee.^41^ In April 2024, Taiwan’s National Health Insurance Administration approved waiving ACP consultation fees for specific groups. Beneficiaries include patients aged 65 and older with major illnesses who meet the conditions for hospice care (e.g., immune diseases, dialysis), mild dementia (Clinical Dementia Rating 0.5-1), difficult-to-treat diseases identified by the Ministry of Health and Welfare, and participants in the Integrated Home Medical Care Plan.^45^ This plan covers individuals homebound due to disabilities or disease, those relying on intensive home medical care or ventilators, and terminally ill patients receiving palliative care.^46^ Expanding beneficiary categories and offering more waiver or co-pay options in the future could promote greater public participation in ACP consultations and AD completion (Table 3).

#### The Inclusiveness of ADs: Addressing Medical, Emotional, and Spiritual Aspects (Section E)

Many U.S. states recognize Five Wishes as a valid AD form, covering personal, emotional, and spiritual aspects of end-of-life care.^47^ Five Wishes is cost-effective, user-friendly, and legally valid in 42 U.S. states, with some requiring notarization, enabling comprehensive documentation of end-of-life care preferences.^47^ It uses plain language, offers 27 languages, and provides an online version to help individuals address medical, personal, emotional, and legal end-of-life wishes.^47,48^ The psychiatric advance directive (PAD) is part of the PSDA,^49^ with 27 states enacting statutes that allow individuals with mental illness to plan future psychiatric care and hospitalizations before crises.^49,50^

In Taiwan, under Article 16 of the PRAA, palliative and supportive care aim to alleviate physical, psychological, and spiritual suffering when ADs to forgo, withdraw, or cease LST or artificial nutrition/hydration are enacted. Medical institutions lacking the capacity to provide palliative care or adequate services should facilitate patient referrals to ensure a dignified death and improved end-of-life care, addressing both physical and emotional needs.^41,51^ ADs forms mainly emphasize medical preferences, often omitting socioemotional and spiritual aspects. This limitation makes it difficult for individuals to articulate personal or psychological preferences for end-of-life care.

Taiwan’s ADs primarily focus on medical aspects, excluding provisions for mental health or psychiatric decisions. This gap limits individuals’ ability to predefine therapies or admissions in cases of future mental incapacity. Legislators and policymakers are urged to expand patient autonomy by incorporating personal, emotional, and spiritual elements into ADs and adding PAD provisions to Taiwan’s PRAA, ACP, and AD processes. These enhancements would provide more comprehensive healthcare planning for end-of-life care (Table 3).

#### Participants Required for Completing an AD (Section F)

Under the PSDA, the AD documents can be filled out by individuals themselves independently or with the assistance of a lawyer.^52^ The AD process does not require the presence of another person, family consent, or medical consultation or certification. Eligibility typically applies to individuals aged 18 and older,^53^ and requirements such as witness or notary presence may vary by state or jurisdiction.^54^

In Taiwan, completing an AD requires at least 1 family member (first or second degree of affinity; PRAA Article 9) to participate in the ACP consultation, unless they are unavailable due to death, being missing, or other exemptions (PRAA Article 9). While this reflects Taiwan’s communal culture, it may create barriers for individuals with strained family relationships or conflicting views on end-of-life care. To enhance patient autonomy, it is recommended that the PRAA be revised to allow extended family members or trusted non-relatives to participate, provided they have no conflicting interests. This would improve the ACP consultation process for AD completion (Table 3).

#### Eligibility Criteria for Healthcare Agents (Section G)

Under the PSDA, there are no restrictions on the relationship between a patient and their healthcare agent. Some states (e.g., North Dakota) may limit certain individuals, such as healthcare or long-term care providers and their employees, from being appointed as healthcare agents.^55^ However, there are no specific restrictions regarding conflicts of interest between the patient and their healthcare agent (e.g., litigators, organ donors, or anyone benefiting from the patient’s death).

Taiwan’s PRAA bars non-heirs from benefiting from a patient’s death, such as being designated as the legatee or recipient of the patient’s remains or organs,^56^ to prevent conflicts but does not restrict healthcare providers as agents, potentially impacting decisions on patient interests.

The PSDA should consider implementing restrictions on the patient-healthcare agent relationship to ensure that healthcare decisions align with the patient’s medical wishes and values. Additionally, we propose that Taiwan’s PRAA extend its current restrictions to include healthcare or long-term care providers, their employees, and affiliates. This would help prevent conflicts of interest that could influence the decision-making of a healthcare agent when needed (Table 3).

#### The Portability of ADs (Section H)

The advancement of technology and cloud-based e-health raises legal concerns about protecting patient privacy and the confidentiality of stored medical records or ADs. This makes it challenging to retrieve and share health-related records, including ADs or ACP documentation, across states and healthcare institutions in the U.S.^57^ Some organizations (e.g., ADVault, Inc., Thorough Care) offer online platforms that allow individuals to upload and share AD forms, such as Five Wishes, Physician Orders for Life-Sustaining Treatment (POLST), Medical Orders for Life-Sustaining Treatment (MOLST), or ACP documents, including audio or video messages, to free online accounts. This approach facilitates the creation, access, updating, and sharing of digital ACP, ADs, or healthcare agent appointments with trusted individuals or healthcare professionals, ensuring timely retrieval of these documents.^58,59^ ADs laws, requirements, validation, and execution vary across states, raising concerns about the portability of ADs during emergencies. Although several solutions have been proposed to ensure ADs are honored across states, many challenges remain unresolved.^60^

A universal or national ADs form may not be feasible, but implementing Five Wishes offers a practical solution for ensuring portability across states.^60^ Recognized in 42 states and D.C., 5 Wishes provides a common framework that supports consistency in ACP nationwide.^60^ Some states, such as Idaho and Maryland, offer an alternative in their ADs laws, prioritizing the honoring or consideration of individuals’ authentic medical wishes rather than strictly validating formal AD documentation.^60^ This approach acknowledges individuals’ healthcare decision-making expressions, allowing their autonomy to align with their culture, attitudes, language, or communication styles.^60^ Further research is needed to improve AD portability across states in critical situations.

On the other hand, under Taiwan’s PRAA, the completed ADs must be registered in the individual’s National Health Insurance Card.^27^ With the patient’s informed consent, the patient’s ADs and medical records are accessible nationwide via cloud in Taiwan. This approach enhances ADs accessibility and portability in urgent situations (Table 3).^61^

#### Strategies for Promoting ADs (Section I)

In the U.S., healthcare facilities participating in Medicare or Medicaid are required to provide written information about patient autonomy in healthcare decision-making and inform patients about their right to complete ADs. This ensures greater awareness and dissemination of AD information.

In contrast, Taiwan relies on voluntary distribution of AD information by healthcare institutions, the government, or the media. To improve communication, it is recommended that healthcare providers in Taiwan proactively discuss ACP or ADs during doctor visits, drawing lessons from the U.S. approach. Physicians or healthcare professionals may also help distribute AD information and arrange or refer their patients to ACP consultations to encourage AD completion.^13^ These approaches aim to enhance awareness, communicate information, and encourage individuals to make end-of-life care arrangements in advance (Table 3).

#### Palliative Care’s Role in ADs (Section J)

In the U.S., palliative care (PC) teams play a significant role in facilitating the completion and promulgation of ADs, thereby enhancing the quality of life for patients and families facing serious illnesses. These teams lead ACP discussions, promoting patient-centered decision-making while navigating the legal and ethical complexities of ADs. They also educate healthcare professionals to ensure ADs are respected across various care settings and provide emotional support to patients and families during end-of-life planning.^62,63^

Similarly, in Taiwan, PC teams are instrumental in fostering the development and completion of ADs, enhancing patients’ quality of life during their final stages. For inpatients with terminal illnesses, medical professionals discuss the patient’s condition with both the patient and their family, offering guidance on the option to consult with the *PC* team. The primary physician arranges consultations with the PC team to help patients develop an AD, particularly concerning end-of-life decisions. For those requiring PC at home, outpatient services are available through a multidisciplinary team of physicians, nurses, social workers, and trained volunteers who regularly visit the patient’s home. Additionally, healthcare institutions provide support from PC social workers, nursing staff, and volunteers to assist the public in completing ADs. In this way, the PC team ensures that patients’ end-of-life wishes are clearly understood and properly documented (Table 3).^64^

## Discussion

The comparative analysis of AD legal frameworks between the U.S. and Taiwan highlights key similarities and differences shaped by each country’s cultural, legal, and healthcare factors. Both countries acknowledge the importance of ADs in end-of-life care, LST options, and respecting autonomous decisions. The shared values highlight their commitment to giving individuals a say in end-of-life care. Cultural and legal variations in AD practice offer opportunities for mutual learning and sharing insights on healthcare and end-of-life policies.

### Required Components: ACP Consultation for Completing an AD

The requirement of an ACP consultation was not stipulated under the PSDA in the U.S. when the Act was implemented in 1991. As awareness and knowledge about ADs could potentially influence how individuals approach their end-of-life care, to influence end-of-life care decisions, the Center for Medicare and Medicaid Services incentivized Medicare/Medicaid providers in 2016 to facilitate voluntary ACP discussions during annual wellness visits, offering reimbursement to support these communications.^36,65^ Some states (e.g., New York) require a healthcare professional’s signature for ADs (e.g., MOLST) to become a legal order,^66^ suggesting an ACP discussion between the patient and the provider. With ACP conversations, patients are more informed and have better access to related education and resources to plan for future care and make mindful and meaningful healthcare choices.^65^

In contrast, the PRAA in Taiwan mandates the implementation of an ACP consultation offered at designated or qualified healthcare institutions, with its seal imprinted on one’s AD documents.^67^ This ACP consultation requirement highlights the importance of individuals’ understanding of ADs and informed decision-making before documenting their medical preferences.

### Incorporating Various Aspects of Care into ADs

In the U.S., various AD or ACP forms, such as POLST, MOLST, DNR orders, and Five Wishes, have different legal potency across states or jurisdictions.^52^ Five Wishes allows individuals to express personal, emotional, and spiritual care preferences alongside medical choices, offering a holistic approach to end-of-life planning.^48,68^ It has been legally recognized in 42 states and has informed ACP and ADs at the public health level nationally and statewide.^47,48^

Conversely, the PRAA in Taiwan currently does not accommodate such inclusive care planning in formalized AD documents. Still, the emotional/spiritual-aspects-incorporated PC is provided when the patient’s AD has been initiated to not perform LST or artificial nutrition/hydration.^51^ Taiwan’s PRAA and AD documents specify medical treatment preferences and the legal processes to complete ADs.^26^ This suggests that the PRAA could expand end-of-life planning to cover more care aspects beyond just medical decisions.

### Involvement of Family Members in the Completion of an AD (Individualism Vs. Collectivism)

In the U.S., while some states may require individuals’ AD documents (e.g., living will form, Five Wishes) to be witnessed or notarized to become lawfully valid, individuals can complete their ADs independently.^52^ In other words, family involvement in the ACP and AD completion processes is not legally required, reflecting a more individualistic approach and stronger assurance of patient autonomy in healthcare and end-of-life planning, compared to Taiwan’s collectivist culture.

The PRAA in Taiwan requires an ACP consultation with the individual, at least one relative/family member (i.e., first or second degree of affinity, unless reported death, missing, or with special reasons), and healthcare professionals before the ADs can be completed.^69^ The involvement of family members in the ACP consultation reflects Taiwan’s Confucian ideology, which emphasizes family order, harmony, collective decision-making, and family-based consent.^70,71,13^ While this ensures loved ones are aware of one’s healthcare wishes and brings diverse perspectives, it may also lead to family conflict, disagreement, or ineffective communication in the ACP consultation. This could undermine the individual’s autonomy, cause emotional distress and distrust in family relationships, and delay AD completion. Additional efforts are needed to address concerns and balance patient autonomy with family involvement.

### Completion Rate of ADs

In the U.S., 80% recognize the value of ADs, but only 37% have completed one since the start of the PSDA in 1991, with an annual increase of 1.1%.^72^ Given the low AD completion rates, addressing potential barriers, such as lack of education, lack of effective physician-patient communication or trusting relationships,^5^ or the denial of mortality,^73^ to allay underlying concerns and encourage ACP and AD completion through policy changes (e.g., amending the PSDA to inquire about AD information to initiate health insurance^74^; providing education, incorporating ACP as part of medical care like cancer screening or dietary counseling to ensure it occurs in our healthcare system)^73,75^ are important.

In Taiwan, cultural shifts and government-promoted online ACP consultations have increased accessibility and AD completion rates. These online consultations allow individuals and families to engage with healthcare professionals remotely, reducing time and travel costs^76^ and potentially increasing AD completion rates. By March 2022, only 33 000 individuals (0.14%) of Taiwan’s 23 million population had completed an AD under the PRAA.^35^ This low completion rate may stem from several factors. First, the PRAA limits ADs to individuals meeting specific conditions like terminal illness, irreversible coma, or severe dementia,^14^ though the list of qualifying conditions, including new ones like congenital multiple joint contracture syndrome added in 2021, is evolving.^31^

Second, the out-of-pocket fee for ACP consultations (USD $100/hour), necessary for AD completion, may deter participation. Although the government waives this fee for certain groups, such as individuals with mild dementia, some argue that charging for consultations ensures individuals value the process, while others believe removing the fee could increase participation.^35^ Third, while the short duration since the PRAA’s implementation in 2019, leaving only about 6 years until 2025, may contribute to the low AD completion rate, other factors, such as limited understanding among healthcare professionals and public misconceptions, such as the belief that completing an AD means refusing all treatment, may also play a role.^35^

Finally, sociocultural factors, such as family-oriented decision-making and attitudes toward death, influence the low AD completion rate. The PRAA’s requirement for family participation in ACP consultations promotes consensus but may also hinder AD completion.^35^ Additionally, the taboo surrounding death in some Taiwanese communities further contributes to reluctance toward end-of-life planning.^35^ Thus, the PRAA’s limitations, financial burdens, and cultural attitudes impact ACP and AD adoption. Efforts should address legal, financial, educational, and cultural barriers while promoting open discussions on end-of-life care.

In sum, the analyzed distinctions show how cultural values and beliefs shape healthcare and legal systems in the U.S. and Taiwan, affecting autonomy in medical decision-making and offering policy implications for end-of-life care.

## Strengths, Limitations, and Future Directions

This study pioneers the analysis and comparison of the legal frameworks regulating patient autonomy in healthcare decision-making between the U.S. (the PSDA) and Taiwan (the PRAA). By highlighting the similarities and differences in the ADs laws of the U.S. and Taiwan, the influence of Eastern and Western cultures on self-determination in health and well-being becomes evident. This cross-cultural learning promotes further research and policy reform, benefiting individuals’ healthcare and well-being.

The review has several limitations. First, dissimilar cultural and societal mechanisms create distinct legal structures in the U.S. and Taiwan, potentially causing bias in law interpretation or overlooking cultural nuances crucial to understanding ADs regulations. Second, laws and policies are shaped by shifts in societal attitudes, leading to changes over time. This study may not capture the latest legislative amendments, court rulings, or policy updates. Third, the PSDA and the PRAA, while sharing similar legislative goals, are influenced by differing healthcare systems and the varying training and values of healthcare professionals, resulting in inconsistent AD implementation. Lastly, social, political, and cultural disparities, along with human subjectivity, could limit the findings’ interpretations and generalizations.

The comparative analysis of the U.S. (the PSDA) and Taiwan (the PRAA) AD legal frameworks offers opportunities for mutual learning, enhancing ACP and ADs awareness and realization. Future research could examine the role of societal and cultural factors in implementing ACP and ADs policies. A discrepancy may exist between patients expressed wishes in ADs and their fulfillment in emergencies. Further research is needed to explore gaps and understand the role of family in ensuring patients’ end-of-life wishes are fulfilled, especially in collectivist, family-oriented societies like Taiwan.^13^ A follow-up study examining how policy implications on end-of-life care have influenced healthcare services, ACP improvements, and AD completion rates would be beneficial.

## Conclusions

The pursuit of dignity and quality of life shapes end-of-life decisions. Advance care planning and directives allow individuals to document healthcare wishes while they remain competent, preparing for situations where communication may be impaired.^6^ U.S. and Taiwan's ACP and ADs policies require healthcare professionals to honor formalized wishes when individuals cannot decide in critical situations. This research enhances education and practices respecting health and death,^6^ safeguards human dignity, and meets the needs of individuals in their final years.

## References

[1] Sabatino C . Advance directives and advance care planning: legal and policy issues. U.S. Department of health and human services assistant secretary for planning and evaluation office of disability, aging and long-term care policy. 2007. https://aspe.hhs.gov/sites/default/files/private/pdf/75366/adacplpi.pdf. Accessed August 25, 2024.

[2] Ott B . Advance directives: the emerging body of research. Am J Crit Care. 1999;8(1):514-519. 9987550

[3] Oulton J Rhodes S Howe C Fain MJ Mohler MJ . Advance directives for older adults in the emergency department: a systematic review. J Palliat Med. 2015;18(6):500-505. doi:10.1089/jpm.2014.0368 25763860

[4] Parkman C Calfee B . Advance directives: honoring your patientʼs end-of-Life wishes. Nursing. 1997;27(4):48-53. doi:10.1097/00152193-199704000-00024 9171666

[5] Prendergast T . Advance care planning: pitfalls, progress, promise. Crit Care Med. 2001;29(2 Suppl):N34-39. doi:10.1097/00003246-200102001-00007 11228571

[6] Prince-Paul M DiFranco E . Upstreaming and normalizing advance care planning conversations-A public health approach. Behav Sci. 2017;7(2):18. doi:10.3390/bs7020018 28417931 PMC5485448

[7] Emanuel L . Advance directives. Annu Rev Med. 2008;59:187-198. doi:10.1146/annurev.med.58.072905.062804 17716024

[8] Wolf S Boyle P Callahan D , et al. Sources of concern about the patient self-determination act. N Engl J Med. 1991;325(23):1666-1671. doi:10.1056/NEJM199112053252334 1944466

[9] Cox D Sachs G . Advance directives and the patient self-determination act. Clin Geriatr Med. 1994;10(3):431-443. 7982160

[10] Heo D Yoo S Keam B Yoo S Koh Y . Problems related to the act on decisions on life-sustaining treatment and directions for improvement. J Hosp Palliat Care. 2022;25(1):1-11. doi:10.14475/jhpc.2022.25.1.1 37674892 PMC10180009

[11] Hsieh W . Virtual reality video promotes effectiveness in advance care planning. BMC Palliat Care. 2020;19(1):125. doi:10.1186/s12904-020-00634-w 32799876 PMC7429729

[12] Patient right to autonomy act, amended 20 january 2021, art 1. Laws & regulations database of the Republic of China (taiwan). https://law.moj.gov.tw/ENG/LawClass/LawAll.aspx?pcode=L0020189. Accessed August 8, 2024.

[13] Wu Y Yang C Lin T , et al. Factors impacting advance decision making and health care agent appointment among Taiwanese urban residents after the passage of patient right to autonomy act. Healthcare. 2023;11(10):1478. doi:10.3390/healthcare11101478 37239764 PMC10217947

[14] Patient right to autonomy act, amended 20 January 2021, art 14. Laws & regulations database of the Republic of China (Taiwan). https://law.moj.gov.tw/ENG/LawClass/LawAll.aspx?pcode=L0020189. Accessed August 8, 2024.

[15] Kucharska W . Tacit knowledge influence on intellectual capital and innovativeness in the healthcare sector: a cross-country study of Poland and the US. J Bus Res. 2022;149:869-883. doi:10.1016/j.jbusres.2022.05.059

[16] Nam S Pritchard K Bae S Hong I . Cross-national comparisons of cognitive and physical health in older adults across China, Japan, and Korea: a systematic review. Inquiry. 2021;58:469580211062451. doi:10.1177/00469580211062451 34898332 PMC8671655

[17] Semlali I Tamches E Singy P Weber O . Introducing cross-cultural education in palliative care: focus groups with experts on practical strategies. BMC Palliat Care. 2020;19(1):171. doi:10.1186/s12904-020-00678-y 33172461 PMC7656760

[18] Park D Eaton T Larson E Palmer H . Implementation and impact of the patient self-determination act. South Med J. 1994;87(10):971-977. doi:10.1097/00007611-199410000-00002 7939924

[19] Teoli D Ghassemzadeh S . Patient self-determination act. In: StatPearls. StatPearls Publishing; 2023. https://www.ncbi.nlm.nih.gov/books/NBK538297/. Accessed August 25, 2024. 30855881

[20] Rep. Levin SM [D M 17]. H.R.4449 - 101st Congress. (1989-1990): to amend titles XVIII and XIX of the Social Security Act to require providers of services and health maintenance organizations under the medicare and medicaid programs to assure that individuals receiving services will be given an opportunity to participate in and direct health care decisions affecting themselves. 1990. https://www.congress.gov/bill/101st-congress/house-bill/4449/text. Accessed August 12, 2024.

[21] What is an advance directive? CaringInfo. https://www.caringinfo.org/planning/advance-directives/what-is-an-advance-directive/. Accessed August 20, 2024.

[22] Hospital sued for wrongful prolongation of life: ethicist offer unique expertise. Med Ethics Advisor. 2021;37(4):37-48.

[23] Locascio L . Wrongful prolongation of life: a new cause of action. Law. 2017. https://www.law.com/njlawjournal/2017/12/04/op-ed-wrongful-prolongation-of-life-a-new-cause-of-action/. Accessed February 17, 2025.

[24] Doctors Hospital of Augusta v . Alicea Administratrix. FindLaw. 2016. https://caselaw.findlaw.com/court/ga-supreme-court/1741593.html. Accessed February 17, 2025.

[25] Pope T . Weisman v. Maryland general hospital, Inc. (Circuit court of Maryland 2016). 2016. https://www.thaddeuspope.com/images/WEISMAN_v_Univ_Maryland_July_2016_resucitate_contra_POLST_.pdf

[26] Patient Right to Autonomy Act . Laws & Regulations Database of The Republic of China (Taiwan). https://law.moj.gov.tw/ENG/LawClass/LawAll.aspx?pcode=L0020189. Accessed August 8, 2024.

[27] Patient right to autonomy act, amended 20 January 2021, art 9. Laws & regulations database of the Republic of China (Taiwan). https://law.moj.gov.tw/ENG/LawClass/LawAll.aspx?pcode=L0020189. Accessed August 8, 2024.

[28] “Enforcement rules of the patient autonomy act” , 2018, Art 11. Laws & Regulations Database of The Republic of China (Taiwan). https://law.moj.gov.tw/LawClass/LawAll.aspx?pcode=L0020199. Accessed August 8, 2024.

[29] “Enforcement rules of the patient autonomy act” , 2018, Art 12. Laws & Regulations Database of The Republic of China (Taiwan). https://law.moj.gov.tw/LawClass/LawAll.aspx?pcode=L0020199. Accessed August 8, 2024.

[30] “Enforcement rules of the patient autonomy act” , 2018, Art 13. Laws & Regulations Database of The Republic of China (Taiwan). https://law.moj.gov.tw/LawClass/LawAll.aspx?pcode=L0020199. Accessed August 8, 2024.

[31] “The Ministry of Health and Welfare Announces the 12th Category of Diseases, Expanding the Clinical Conditions Applicable under the Patient Autonomy Act” [in Chinese]. Ministry of Health and Welfare. 2021. https://www.hospice.org.tw/content/3408. Accessed August 12, 2024.

[32] “The Ministry of Health and Welfare Has Announced 11 Types of Diseases to Expand the Clinical Conditions Applicable under the Patient Self-Determination Act” [in Chinese]. Ministry of Health and Welfare. 2020. https://www.mohw.gov.tw/cp-16-50877-1.html. Accessed August 22, 2024.

[33] Patient right to autonomy act, amended 20 January 2021, art 3, Section 1. Laws & regulations database of the Republic of China (Taiwan). https://law.moj.gov.tw/ENG/LawClass/LawAll.aspx?pcode=L0020189. Accessed August 8, 2024.

[34] Patient right to autonomy act, amended 20 January 2021, art 3, section 2. Laws & regulations database of the Republic of China (Taiwan). https://law.moj.gov.tw/ENG/LawClass/LawAll.aspx?pcode=L0020189. Accessed August 8, 2024.

[35] Tsai DFC . The law and practice of advance directives in Taiwan. In: Cheung D Dunn M , eds. Advance Directives across Asia: A Comparative Socio-Legal Analysis. doi:10.1017/9781009152631.006. Cambridge University Press; 2023:75-89.

[36] FAQs . Advance care planning under medicare. Medicare pays advance care planning. 2017. https://polst.org/wp-content/uploads/2018/01/2017.11-Advance-Care-Planning-Under-Medicare-CMS-ACP-Codes-FAQs-CCCC.pdf. Accessed 15 August 2024.

[37] Chang T . “A comparative study on the characteristics of advance care planning consultations between Taiwan and the United States” [in Chinese]. Law Monthly. 2018;69(5):59-74. doi:10.6509/TLM.201805_69(5).0003

[38] Gordon M Le T Kabir F . Telehealth advance care planning cost effectiveness. Arch Clin Med Case Rep. 2023;7(2):171-177. doi:10.26502/acmcr.96550595

[39] Powers J Abraham L Tennessee Valley Healthcare System, Nashville, Tennessee 37232, USA . Outpatient–focused advance care planning: telehealth consultation for geriatric primary care patients. Palliat Med Hosp Care Open J. 2020;6(1):1-4. doi:10.17140/PMHCOJ-6-133

[40] Watts K Gazaway S Malone E , et al. Community tele-pal: a community-developed, culturally based palliative care tele-consult randomized controlled trial for African American and white rural southern elders with a life-limiting illness. Trials. 2020;21(1):672. doi:10.1186/s13063-020-04567-w 32703245 PMC7376880

[41] Hu IT Hsu MY Hsueh KC Chen JI . A brief discussion on the differences between the patient right to autonomy act and the hospice and palliative care act. Family Medicine and Primary Medical Care. 2020;35(9):256-262.

[42] Yen C Lin C Su Y , et al. The characteristics and motivations of Taiwanese people toward advance care planning in outpatient clinics at a community hospital. Int J Environ Res Publ Health. 2021;18(6):2821. doi:10.3390/ijerph18062821 PMC7999986 33802074

[43] Patient right to autonomy act, amended 20 January 2021, art 9, section 3. Laws & regulations database of the Republic of China (Taiwan). https://law.moj.gov.tw/ENG/LawClass/LawAll.aspx?pcode=L0020189. Accessed August 8, 2024.

[44] Civil code, amended 20 January 2021, art 968. Laws & regulations database of the Republic of China (Taiwan). https://law.moj.gov.tw/ENG/LawClass/LawAll.aspx?pcode=B0000001. Accessed August 8, 2024.

[45] National Health Insurance Coverage for Advance Care Planning Consultation Fees. National Health Insurance Administration, Ministry of Health and Welfare. 2024. https://www.nhi.gov.tw/ch/dl-71885-c73955ff6ac44882873afcff94c9a924-1.pdf. Accessed February 16, 2025.

[46] National Health Insurance Integrated Home Medical Care Plan. National Health Insurance Administration, Ministry of Health and Welfare. 2023. https://www.nhi.gov.tw/ch/cp-5195-2e804-2875-1.html. Accessed August 11, 2024.

[47] Atherton K . Project five wishes: promoting advance directives in primary care. J Am Assoc Nurse Pract. 2020;32(10):689-695. doi:10.1097/JXX.0000000000000289 31567780

[48] Five Wishes . Five wishes. https://www.fivewishes.org/. Accessed August 10, 2024.

[49] A practical guide to psychiatric advance directives. Substance Abuse and Mental Health Services Administration. 2019. https://store.samhsa.gov/sites/default/files/psychiatric-advance-directives-pep19-pl-guide-4.pdf. Accessed August 20, 2024.

[50] CMS Manual System Pub. 100-07 State Operations Provider Certification. Transmittal 1. Department of Health & Human Services Centers for Medicare & Medicaid Services. 2004. https://www.cms.gov/Regulations-and-Guidance/Guidance/Transmittals/downloads/r1som.pdf. Accessed August 11, 2024.

[51] Patient right to autonomy act, amended 20 January 2021, art 16. Laws & Regulations Database of the Republic of China (Taiwan). https://law.moj.gov.tw/ENG/LawClass/LawAll.aspx?pcode=L0020189. Accessed August 8, 2024.

[52] Advance Care Planning . Advance Directives for Health Care. National Institute on Aging; 2022. https://www.nia.nih.gov/health/advance-care-planning/advance-care-planning-advance-directives-health-care#find. Accessed 21 August 2024.

[53] Directives A . Nationwide children’s hospital. 2013. https://www.nationwidechildrens.org/family-resources-education/health-wellness-and-safety-resources/helping-hands/advance-directives. Accessed 20 August 2024.

[54] Rolnick J Shea J Hart J Halpern S . Patients’ perspectives on approaches to facilitate completion of advance directives. Am J Hosp Palliat Care. 2019;36:526-532. doi:10.1177/1049909118824548 30696253

[55] Chapter 23-06. 5 Health Care Directives . North Dakota century code t23c06.5. https://ndlegis.gov/cencode/t23c06-5.pdf. Accessed 21 August 2024.

[56] Patient right to autonomy act, amended 20 January 2021, art 10. Laws & regulations database of the Republic of China (Taiwan). https://law.moj.gov.tw/ENG/LawClass/LawAll.aspx?pcode=L0020189. Accessed August 8, 2024.

[57] Medows AM. Legal and policy aspects of the intersection between cloud computing and the U.S. healthcare industry. *Jolt Digest**. *Published September 11, 2015. Accessed August 20, 2024. https://jolt.law.harvard.edu/digest/legal-and-policy-aspects-of-the-intersection-between-cloud-computing-and-the-u-s-healthcare-industry

[58] MyDirectives Team . ADVault changes name to MyDirectives, unveils revamped web App and platform. myDirectives. 2024. https://www.mydirectives.com/press-releases/advault-changes-name-to-mydirectives-unveils-revamped-web-application-and-platform. Accessed 9 August 2024.

[59] Coordinate care for end-of-life values. *Thorough Care*. 2024. Accessed August 20, 2024. https://www.thoroughcare.net/advance-care-planning

[60] Sabatino C . Can my advance directives travel across state lines? An essay on portability. Bifocal. 2016;38(1):3-7.

[61] Liu TJ Lee HT Wu F . Building an electronic medical record system exchanged in FHIR format and its visual presentation. Healthcare. 2023;11(17):2410. doi:10.3390/healthcare11172410 37685442 PMC10486699

[62] ANA Center for Ethics and Human Rights . Nurses’ Roles and Responsibilities in Providing Care and Support at the End of Life. American Nurses Association; 2024. https://www.nursingworld.org/globalassets/docs/ana/practice/official-position-statements/nursesrolesandresponsibilitiesinprovidingcareandsupportattheendoflife_revised_bod-approved_final.pdf

[63] Leung DYP Chung JOK Chan HYL , et al. Effects of a structured, family-supported, and patient-centred advance care planning on end-of-life decision making among palliative care patients and their family members: protocol of a randomised controlled trial. BMC Palliat Care. 2024;23(1):257. doi:10.1186/s12904-024-01588-z 39511666 PMC11542196

[64] Helpline for Palliative Care Consultation Services. Ministry of Health and Welfare. 2013; https://www.mohw.gov.tw/cp-3210-23576-1.html. Accessed February 17, 2025.

[65] Brull J . Advance care planning: how to have the conversation you want with your patients. Fam Pract Manag. 2019. https://www.aafp.org/pubs/fpm/issues/2019/1100/p18.html. Accessed 10 August 2024. 31714046

[66] Medical Orders for Life-Sustaining Treatment (MOLST) . New York State Department of Health; 2023. https://www.health.ny.gov/professionals/patients/patient_rights/molst/. Accessed 15 August 2024.

[67] Patient right to autonomy act, amended 20 January 2021, art 9, section 1. Laws & regulations database of the Republic of China (Taiwan). https://law.moj.gov.tw/ENG/LawClass/LawAll.aspx?pcode=L0020189. Accessed August 8, 2024.

[68] Francoeur R Burke N Wilson A . The role of social workers in spiritual care to facilitate coping with chronic illness and self-determination in advance care planning. Soc Work Publ Health. 2016;31(5):453-466. doi:10.1080/19371918.2016.1146199 27187806

[69] Patient Right to Autonomy Act, Amended 20 January 2021, art 9, Section 2 . Laws & regulations database of the Republic of China (Taiwan). https://law.moj.gov.tw/ENG/LawClass/LawAll.aspx?pcode=L0020189. Accessed August 8, 2024.

[70] Badanta B González-Cano-Caballero M Suárez-Reina P Lucchetti G de Diego-Cordero R . How does confucianism influence health behaviors, health outcomes and medical decisions? a scoping review. J Relig Health. 2022;61(4):2679-2725. doi:10.1007/s10943-022-01506-8 35141796 PMC9314298

[71] Lee S . Intimacy and family consent: a confucian ideal. J Med Philos. 2015;40(4):418-436. doi:10.1093/jmp/jhv015 26142440

[72] Aaron S Musacchio C Douglas S . Understanding factors that predict advance directive completion. Palliat Med Rep. 2022;3(1):220-224. doi:10.1089/pmr.2021.0073 36876293 PMC9983130

[73] Fried T Drickamer M . Garnering support for advance care planning. JAMA. 2010;303(3):269-270. doi:10.1001/jama.2009.1956 20085956 PMC2899482

[74] Eiser A Weiss M . The underachieving advance directive: recommendations for increasing advance directive completion. Am J Bioeth. 2001;1(4):W10. 12861997

[75] Hickman S Hammes B Moss A Tolle S . Hope for the future: achieving the original intent of advance directives. Hastings Cent Rep. 2005;Spec No:S26-30. doi:10.1353/hcr.2005.0093 16468252

[76] Encouraging Online Advance Care Planning Consultation Clinics to Improve the Accessibility of Advance Directives. Ministry of Health and Welfare. 2023. https://www.mohw.gov.tw/cp-6568-73103-1.html. Accessed August 22, 2024.

